# Pharmacogenomics and clinical response to antipsychotic treatment. Expectations vs reality.

**DOI:** 10.1192/j.eurpsy.2023.2151

**Published:** 2023-07-19

**Authors:** N. Papadimitriou, D. Antoniadis

**Affiliations:** 1 4th PICU, Mental Health Hospital of Thessaloniki; 2 School of Medicine, Aristotle University of Thessaloniki, Thessaloniki, Greece

## Abstract

**Introduction:**

According to the ICD-10 classification system, manic episodes in bipolar affective disorder (BAD) are typically characterised by sudden onset of symptoms and a duration between two weeks and five months. Mood stabilizers and 2^nd^ generation antipsychotics are recommended as first-line treatment. Herein we report a young individual with Bipolar Affective Disorder (BAD), who had an unexpected response to medication given his pharmacogenomic results.

**Objectives:**

To investigate the clinical use of the pharmagenomic results of antipsychotic treatment.

**Methods:**

Clinical assessment, psychometric evaluation and pharmacogenomic analysis.

**Results:**

Cerebral CT scanning showed dilatation of the ventricular system and subarachnoid spaces, findings that are not compatible with the patient’s age, but are seen in individuals with BAD (Keener & Phillips, 2007).

For the purposes of psychological evaluation, her underwent the psychometric assessments Rorschach and MMPI. Rorschach evaluation showed mild manic traits with grandiose ideas and dissocial personality traits through hetero-catastrophic ideas. The MMPI evaluation indicated a psychopathic personality with borderline traits. His clinical examination and psychiatric history confirmed the diagnosis of BAD.

In order to investigate the patient’s poor response to prior pharmacological treatment and determine the future optimal, we referred him for pharmacogenomic testing. The latter involved determination of allele frequencies predicting variations in activity of cytochrome (CYP) P450 drug metabolizing enzymes. Genotyping of CYP450 is known to have a clinical impact on treatment choice and dosage adjustment in patients with BAD (Yenilmez, Tamam, Karaytug & Tuli, 2018). Based on his results, he was discharged on aripiprazole.

He scored 44 in YMRS (Young Mania Rating Scale) upon admission. Blood tests were normal and no other health problems were evident.

Twenty days later, the patient was re-admitted due to clinical deterioration, which prompted the replacement of aripiprazole with olanzapine. He responded satisfactorily to olanzapine and was discharged in good condition on a dosage of 10mg OD and amp 405mg once/month. He continued his treatment with valproic acid 2000mg daily.

**Conclusions:**

The patient responded well to olanzapine, which is strongly related to the CYP1A2 enzyme. Based on the prediction that he would be a rapid metabolizer, olanzapine should only have been effective at higher doses. Besides, the patient was a smoker, meaning he should have required even higher doses, as smoking induces the CYP1A2 enzyme.

**Disclosure of Interest:**

None Declared

